# Generation and characterisation of seven induced pluripotent stem cell lines from two patients with Parkinson’s disease carrying the pathological variant c.1087G>T of the LGR4 gene

**DOI:** 10.18699/vjgb-25-03

**Published:** 2025-02

**Authors:** V.S. Podvysotskaya, E.V. Grigor’eva, A.A. Malakhova, J.M. Minina, Y.V. Vyatkin, E.A. Khabarova, J.A. Rzaev, S.P. Medvedev, L.V. Kovalenko, S.M. Zakian

**Affiliations:** Institute of Cytology and Genetics of the Siberian Branch of the Russian Academy of Sciences, Novosibirsk, Russia Novosibirsk State University, Novosibirsk, Russia; Institute of Cytology and Genetics of the Siberian Branch of the Russian Academy of Sciences, Novosibirsk, Russia Institute of Chemical Biology and Fundamental Medicine of the Siberian Branch of the Russian Academy of Sciences, Novosibirsk, Russia; Institute of Cytology and Genetics of the Siberian Branch of the Russian Academy of Sciences, Novosibirsk, Russia Institute of Chemical Biology and Fundamental Medicine of the Siberian Branch of the Russian Academy of Sciences, Novosibirsk, Russia; Institute of Cytology and Genetics of the Siberian Branch of the Russian Academy of Sciences, Novosibirsk, Russia; NOVEL Ltd., Novosibirsk, Russia; Institute of Cytology and Genetics of the Siberian Branch of the Russian Academy of Sciences, Novosibirsk, Russia Federal Neurosurgical Center of the Ministry of Health of the Russian Federation, Novosibirsk, Russia; Federal Neurosurgical Center of the Ministry of Health of the Russian Federation, Novosibirsk, Russia; Institute of Cytology and Genetics of the Siberian Branch of the Russian Academy of Sciences, Novosibirsk, Russia Institute of Chemical Biology and Fundamental Medicine of the Siberian Branch of the Russian Academy of Sciences, Novosibirsk, Russia; Surgut State University, Surgut, Khanty-Mansiysk Autonomous Okrug – Ugra, Russia; Institute of Cytology and Genetics of the Siberian Branch of the Russian Academy of Sciences, Novosibirsk, Russia Institute of Chemical Biology and Fundamental Medicine of the Siberian Branch of the Russian Academy of Sciences, Novosibirsk, Russia

**Keywords:** Parkinson’s disease, reprogramming, induced pluripotent stem cells, LGR4 gene, болезнь Паркинсона, репрограммирование, индуцированные плюрипотентные стволовые клетки, ген LGR4

## Abstract

Parkinson’s disease is a neurodegenerative disorder affecting dopaminergic neurons of the substantia nigra pars compacta. The known pathological genetic variants may explain the cause of only 5 % of cases of the disease. In our study, we found two patients with a clinical diagnosis of Parkinson’s disease with the genetic variant c.1087G>T (p.Gly363Cys) of the LGR4 gene. The LGR4 gene encodes the membrane receptor LGR4 (leucine rich repeat containing G protein-coupled receptor 4) associated with the G protein. We hypothesize that the LGR4 gene may be either a direct cause or a risk factor for this disease, since it is one of the main participants of the WNT/β-catenin signalling pathway. This signalling pathway is necessary for the proliferation of neurons during their differentiation, which may lead to Parkinson’s disease. To study the relationship between this genetic variant and Parkinson’s disease, an ideal tool is a cellular model based on induced pluripotent stem cells (iPSCs) and their differentiated derivatives, dopaminergic neurons. We reprogrammed the peripheral blood mononuclear cells of the two patients with the c.1087G>T variant of the LGR4 gene with non-integrating episomal vectors expressing OCT4, SOX2, KLF4, LIN28, L-MYC and mp53DD proteins. The obtained seven lines of induced pluripotent stem cells were characterised in detail. The iPSCs lines obtained meet all the requirements of pluripotent cells, namely, they stably proliferate, form colonies with a morphology characteristic of human pluripotent cells, have a normal diploid karyotype, express endogenous alkaline phosphatase and pluripotency markers (OCT4, NANOG, SSEA-4 and SOX2) and are capable to differentiate into derivatives of the three germ layers. The iPSC lines obtained in this work can be used as a tool to generate a relevant model to study the effect of the pathological variant c.1087G>T of the LGR4 gene on dopaminergic neuron differentiation.

## Abstract (ru)

Болезнь Паркинсона – тяжелое нейродегенеративное заболевание, поражающее дофаминергические нейроны компактной части черной субстанции головного мозга. Известные патологические генетические варианты, ассоциированные с болезнью Паркинсона, объясняют причину всего 5 % случаев заболевания, поэтому исследования в этой области актуальны и активно продолжаются. В данном исследовании мы обнаружили двух пациентов с клиническим диагнозом «болезнь Паркинсона» с генетическим вариантом LGR4:с.1087G>T (p.Gly363Cys, rs117543292). Этот ген кодирует мембранный рецептор LGR4 (leucine rich repeat соntаining G protein-coupled receptor 4), ассоциированный с G-белком, который участвует в регуляции функционирования сигнального пути WNT/β-катенин. Данный сигнальный путь необходим для пролиферации нейронов во время их дифференцировки, поэтому его дисфункция в результате гетерозиготной мутации с.1087G>T в гене LGR4 нарушает дифференцировку дофаминергических нейронов, что может приводить к болезни Паркинсона. Идеальным инструментом для изучения связи этого генетического варианта с болезнью Паркинсона является клеточная модель на основе индуцированных плюрипотентных стволовых клеток (ИПСК) и их дифференцированных производных – дофаминергических нейронов. В результате репрограммирования эписомными векторами, экспрессирующими белки OCT4, SOX2, KLF4, LIN28, L-MYC и mp53DD, мононуклеарных клеток периферической крови двух пациентов с вариантом с.1087G>T гена LGR4 нами были получены и детально охарактеризованы семь линий ИПСК. Данные ИПСК отвечают всем требованиям плюрипотентных клеток, а именно: стабильно пролиферируют, образуют колонии с характерной для плюрипотентных клеток человека морфологией, имеют нормальный диплоидный кариотип, экспрессируют щелочную фосфатазу и маркеры плюрипотентности (OCT4, NANOG, SSEA-4 и SOX2) и способны дифференцироваться в производные трех зародышевых листков – энто-, экто- и мезодерму. Полученные в работе линии ИПСК будут в дальнейшем использованы для создания релевантной модели, направленной на исследование эффекта варианта с.1087G>T гена LGR4 на развитие патологического фенотипа дофаминергических нейронов.

## Introduction

Parkinson’s disease is the second most prevalent neurodegenerative disorder after Alzheimer’s disease (Wang et al., 2020). It is characterised by the degeneration of dopaminergic neurons in the substantia nigra, which are responsible for regulating movement. The resulting symptoms include tremor, bradykinesia and rigidity. The destruction of these neurons can be attributed to various factors. These include accumulation of alpha-synuclein protein and formation of Lewy bodies, dysfunction of mitochondria and lysosomes, problems of synaptic and vesicle transport. These factors, when combined, lead to an accelerated death of neurons. Some of the signs of Parkinson’s disease at the cellular level are oxidative stress resulting from mitochondrial dysfunction (Niu et al., 2021) and endoplasmic reticulum (ER) stress resulting from the accumulation of large amounts of misfolded proteins (Fernandes et al., 2016; Marciniak et al., 2022).

It is currently understood that more than 20 genes are associated with Parkinson’s disease, the most prevalent mutations of which are found in the GBA1, LRRK2, PRKN, and PINK1 genes (Funayama et al., 2023). However, it has been determined that only approximately 5 % of cases of Parkinson’s disease are attributable to the inheritance of one of the genes associated with the disease, with the remaining 16–36 % being manifested as a result of non-monogenic inheritance. It can thus be concluded that research into genetic variants associated with the onset of Parkinson’s disease remains an active area of study

Following a comprehensive analysis of the exomic sequencing results for a cohort of over 70 patients diagnosed with Parkinson’s disease, who are under observation at the Federal Neurosurgical Center of the Ministry of Health of the Russian Federation (Novosibirsk), it was found that two patients of different genders, who are not related, carry the c.1087G>T variant of the LGR4 gene (SRA databases, project PRJNA563295). There are currently 48 known single nucleotide substitutions in this gene, three of which are pathogenic missense mutations c.2531A>G (p.Asp844Gly), c.286A>G (p.Ile96Val) and c.1087G>T (p.Gly363Cys) (Mancini et al., 2023).

LGR4 is a G-coupled receptor that plays a role in the function of the WNT/β-catenin and cAMP/protein kinase A signalling pathways (Shi et al., 2021). Previously, it was shown that the c.1087G>T variant of the LGR4 gene is pathogenic and leads to delayed puberty due to a violation of the WNT/β-catenin signalling pathway (Mancini et al., 2023), which is also involved in embryogenesis in the development of tissues and organs such as the gonads, kidneys, nervous system, liver and others. It should be noted that this pathway is also required for the development and differentiation of dopaminergic neurons (Marchetti et al., 2020). This correlation, together with the involvement of the LGR4 protein in the WNT/β-catenin signalling pathway, leads us to speculate that the genetic variant LGR4:c.1087G>T may cause dysfunction or death of dopaminergic neurons, possibly leading to Parkinson’s disease. To study the association of this genetic variant with pathogenic processes leading to the death of dopaminergic neurons, the first step is to create a cellular model based on induced pluripotent stem cells (iPSCs) and relevant cell types, dopaminergic neurons, differentiated from them.

The pluripotent state of iPSCs is maintained by adding basic fibroblast growth factor (FGF-basic/bFGF) to the culture medium. To confirm the characteristics of pluripotency and self-renewal of cells, various tests are performed (Grigor’eva et al., 2023), such as qualitative (immunofluorescence staining) and quantitative (real-time PCR) analyses of the expression levels of various pluripotency factors (NANOG, SSEA-4, OCT4, SOX2, TRA1-81, TRA1-60, etc.). It is known that various karyotype abnormalities (polyploidisation, translocations, deletions, aneuploidies, etc.) are possible during prolonged in vitro cell culture, so karyotype analysis is one of the key tests for characterising the obtained iPSC lines. The most important test of cell pluripotency is spontaneous differentiation, which is necessary to confirm the ability of iPSCs to give rise to cell derivatives of all three germ layers (ecto-, ento- and mesoderm). In addition, the following shall be performed to characterise the iPSC lines obtained: STR analysis to confirm the origin of the iPSC lines obtained from a peripheral blood mononuclear cell (PBMC) donor; tests to indicate the presence of genetic variants of the PBMC donor in the iPSCs or to confirm the presence of genetic modifications in the case of transgenesis of iPSCs; PCR to eliminate exogenous DNA/ episomes that enabled somatic cell reprogramming; and routine tests to ensure the absence of intra-laboratory contamination.

The capacity of iPSCs to yield derivatives of a virtually limitless range of cell types, including neurons, cardiomyocytes, liver cells, endothelial cells and numerous others, renders them a promising tool for generating cell models and investigating the pathogenesis of various inherited diseases in relevant cell types. For instance, by reprogramming PBMCs, culturing the resulting iPSCs and further differentiating them into dopaminergic neurons (Grigor’eva et al., 2023) and astrocytes (Yarkova et al., 2023), it will be possible to obtain in vitro models of Parkinson’s disease. This platform will facilitate the analysis of the contribution of the genetic variant LGR4:c.1087G>T to Parkinson’s disease development, as well as the study of its molecular and genetic pathogenesis. It will also support the exploration of new drug targets within diverse signalling pathways, and the prevention of pathogenic processes that result in dopaminergic neuron death. Furthermore, it will enable the evaluation of potential medical drugs.

## Materials and methods

Ethics Statement. The study was approved by the Research Ethics Committee of the Federal Neurosurgical Center of the Ministry of Health of the Russian Federation (Novosibirsk), Protocol No. 1, dated 14 March 2017. Peripheral blood samples from patients were provided by the Federal Neurosurgical Center. Patients signed a voluntary informed consent and an information sheet.

Isolation of PBMCs. PBMCs were isolated in a Ficoll gradient and frozen as previously described (Grigor’eva et al., 2024b).

Reprogramming of patient-specific PBMCs. Reprogramming of PBMCs was performed by transfection with episomal vectors expressing SOX2, OCT4, KLF4, L-MYС, LIN28 and the dominant negative form of mouse p53 protein – mp53DD (Addgene ID No. 41813–14, 41855–57) according to the previously described method (Grigor’eva et al., 2023). Transfection was performed using a Neon Transfection System (Thermo Fisher Scientific), programme: 1,650 V, 10 ms, 3 times. Primary colonies of iPSCs were replated into 1 cm2 wells previously coated with mouse embryonic fibroblasts (MEF) inactivated with mitomycin C (Sigma-Aldrich).

Cultivation of patient-specific iPSCs. iPSCs were cultured on MEF as previously described (Yarkova et al., 2024). Colonies of iPSCs were disaggregated using TrypLE Express (Thermo Fisher Scientific) and plated once every 3–4 days at a 1:10 ratio with the addition of a ROCK inhibitor, 2 μM thiazovivin (Sigma-Aldrich).

Histochemical detection of endogenous alkaline phosphatase. Alkaline phosphatase was detected using the SIGMAFAST™ BCIP®/NBT kit (Sigma-Aldrich). The result was analysed using a Nikon Eсlipse Ti-E microscope (Nikon) with NIS Elements Advanced Research software version 4.30.

Immunofluorescence staining. Immunofluorescence staining was performed according to the previously described method (Grigor’eva et al., 2024a). The preparations were analysed on a Nikon Eclipse Ti-E microscope using NIS Elements Advanced Research version 4.30 software. The list of antibodies used is shown in Table 1.

**Table 1.**
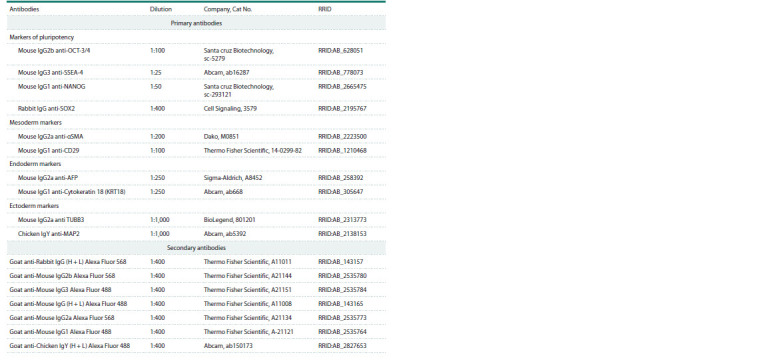
List of antibodies used in the work

Spontaneous differentiation of iPSCs. To determine the potential of the resulting iPSC lines, spontaneous differentiation was performed by the formation of embryoid bodies followed by immunofluorescence staining. iPSCs were grown on MEF in Petri dishes (20 cm2) to a density of 80–90 %, after which the cells were incubated in 0.15 % collagenase type IV (Thermo Fisher Scientific) for 20–40 min. The iPSC colonies were then carefully pipetted to separate them from the feeder cells, centrifuged at 100 g for 5 min, the supernatant was decanted, suspended in iPSC growth medium without bFGF, and the colonies were transferred to a Petri dish (20 cm2) coated with 1 % agarose. After 14 days, embryoid bodies obtained from the colonies were plated on 8-well Chambered Coverglass plates (Thermo Fisher Scientific) coated with Matrigel. After 7–9 days, they were fixed in 4 % paraformaldehyde (Sigma-Aldrich) for 10 min and immunofluorescence analysis was performed for markers of the three germ layers. The list of antibodies is given in Table 1.

Karyotyping of iPSC lines. Karyotype analysis was performed using a previously developed and described method (Yarkova et al., 2023).

Isolation of genomic DNA and RNA. DNA was isolated using QuickExtract™ DNA extraction solution (Lucigen) according to the manufacturer’s protocol. For RNA isolation, cells were grown to an area of 8–12 cm2, lysed in 0.5–1 ml TRIzol Reagent (Thermo Fisher Scientific), and RNA was isolated according to the manufacturer’s protocol. RNA concentration was determined using an EzDrop 1000C spectrophotometer (Blue-Ray Biotech). cDNA synthesis was performed using M-MuLV–RH reverse transcriptase (Biolabmix).

Detection of genetic variants in PBMCs and iPSCs. Sequencing of the patients’ exome was performed at Genoanalitika LLC, Moscow. Genomic DNA isolated from PBMCs was fragmented into 300 nucleotide pair fragments using ultrasound on a Covaris S2. Genomic DNA (800 ng) was used to prepare a library using the NEBNext® Ultra™ II DNA Library Prep Kit for Illumina (New England Biolabs) and Sure Select AllExome V7 (Agilent). Library sequencing was performed on a HiSeq 2500 (Illumina) with paired reads of 150 nucleotides from both ends. Raw exome sequencing results are available in the SRA database (project PRJNA563295, samples SAMN42050732, https://www.ncbi.nlm.nih.gov/biosample/42050732 (PD58), SAMN42050755, https://www.ncbi.nlm.nih.gov/ biosample/42050755 (PD69)).

The presence of the c.1087G>T variant (rs117543292) of the LGR4 gene was determined by Sanger sequencing of the obtained iPSC lines and patient-derived PBMCs (PD58 and PD69) using primers for the LGR4 gene (Table 2). DNA from a conditionally healthy donor was used as a control. Reactions were performed on a T100 thermal cycler (Bio-Rad) using a BioMaster HS-Taq PCR-Color (2×) (Biolabmix) with the following program: 95 °C for 3 min; 35 cycles: 95 °C for 30 s, 65 °C for 30 s, 72 °C for 30 s; and 72 °C for 5 min. Sequencing reactions were performed using the Big Dye Terminator V.3.1. Cycle Sequencing Kit (Applied Biosystems) and analysed on an ABI 3130XL Genetic Analyzer at the Genomics Core Facility SB RAS (http://www.niboch. nsc.ru/doku.php/sequest).

**Table 2.**
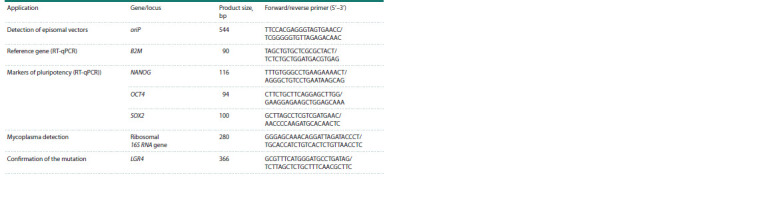
List of primers used in the work

PCR analysis for detection of reprogramming episomes and mycoplasma contamination. PCR was performed using BioMaster HS-Taq PCR-Color (2×) (Biolabmix) on a T100 thermal cycler (Bio-Rad), programme: 95 °C for 5 min; 35 cycles: 95 °C for 15 s, 60 °C for 15 s, 72 °C for 20 s. DNA fragments of Mycoplasma spp. from the BioMaster Myco-visor kit for detection of mycoplasma by RT-PCR (Biolabmix) were used as a positive control. Negative control was H2O. Primers are listed in Table 2. After electrophoresis, the result was visualised under ultraviolet light using the Gel Doc XR+ System (Bio-Rad).

Quantitative RT-PCR for pluripotency markers. Quantitative PCR (RT-qPCR) was performed on a LightCycler 480 II system (Roche) using BioMaster HS-qPCR SYBR Blue 2× (Biolabmix) with the following programme: 95 °C for 5 min; 40 cycles: 95 °C for 10 s, 60 °C for 1 min. The embryonic stem cell line HUES9 (HVRDe009-A) was used as a positive control for the expression of pluripotency markers (Cowan et al., 2004). The pluripotency marker primers used in this work are listed in Table 2. The results were processed using the ΔΔCT method (Livak, Schmittgen, 2001).

STR analysis. The genetic material of the obtained iPSC lines and PBMCs from patients PD58 and PD69 was sent to Genoanalitika LLC (Moscow) for STR analysis. The STR analysis was performed for 26 loci using the COrDIS EXPERT 26 reagent kit (Russia).

## Results

Obtaining and characterisation of the cell lines

Exomic sequencing analysis of early onset Parkinson’s disease patients at the Federal Neurosurgical Center of the Ministry of Health of the Russian Federation (Novosibirsk) revealed two individuals (a 37-year-old woman (PD58) and a 66-year-old man (PD69)) with the variant LGR4:c.1087G>T (rs117543292).

PBMCs were isolated from patients’ peripheral blood and then reprogrammed to revert to a pluripotency state, using episomal vectors encoding the pluripotency factors OCT4, SOX2, KLF4, LIN28, L-MYC, and the dominant-negative form of the mouse p53 protein, mp53DD (Okita et al., 2013). In this study, 21 cell lines of PD58 and 10 cell lines of PD69 were generated. Primary analysis for the presence/ absence of episomal vectors and karyotyping allowed us to select three lines from the first patient (ICGi053-A/PD58-4, ICGi053-B/ PD58-7 and ICGi053-C/PD58-14) and four lines from the second one (ICGi054-A/PD69-1/1, ICGi054-B/PD69-2/1, ICGi054-C/PD69-4 and ICGi054- D/PD69-5). All the lines were registered in the Human Pluripotent Stem Cell Registry (hPSCreg, https://hpscreg.eu). Information on cell lines can be found in hPSCreg at the following links: https:// hpscreg.eu/cell-line/ICGi053-A, https://hpscreg.eu/cell-line/ ICGi053-B, https://hpscreg.eu/cell-line/ICGi053-C, https:// hpscreg.eu/cell-line/ICGi054- A, https://hpscreg.eu/cell-line/ ICGi054-B, https://hpscreg.eu/cell-line/ICGi054-C и https:// hpscreg.eu/cell-line/ICGi054-D.

**Fig. 1.**
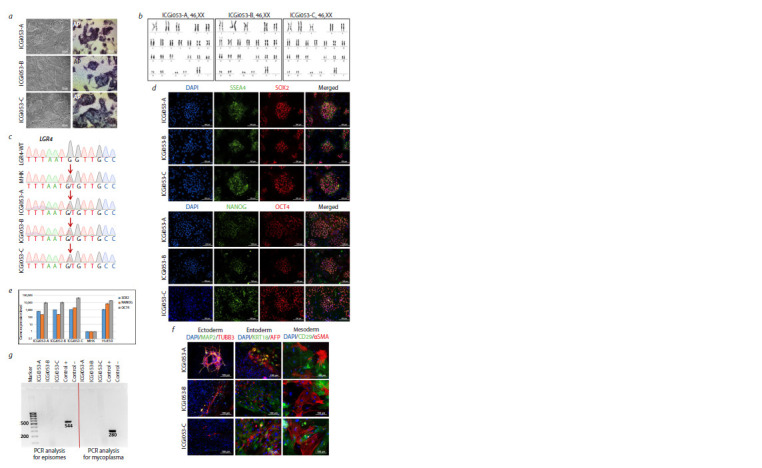
Characterisation of three iPSC lines ICGi053-A, ICGi053-B and ICGi053-C. a – colony morphology and detection of alkaline phosphatase (AP) in iPSCs; b – karyotyping of iPSC lines using DAPI banding; c – sequenograms of PCR products obtained from genomic DNA of a patient with early onset Parkinson’s disease PD58, a healthy donor (LGR4-WT), iPSC lines and PBMCs corresponding to positions 27377174-27377186 on human chromosome 11 (GRCh38 genome assembly). The position of the gene variant studied is LGR4 NC_000011.10:g.27377180C>A. The nucleotide substitution LGR4:c.1087G>T is indicated by the red arrow; d – immunofluorescence staining for markers of pluripotency: NANOG (green), OCT4 (red), SSEA-4 (green), SOX2 (red); nuclei stained with DAPI (blue); e – quantitative RT-PCR for pluripotency markers (OCT4, SOX2, NANOG) of derived iPSC lines, patient PBMCs and human embryonic stem cell line HUES9; f – immunofluorescence staining of spontaneously differentiated cells for markers of ectoderm (TUBB3 (red), MAP2 (green)), entoderm (keratin 18/KRT18 (green), AFP (red)) and mesoderm (αSMA (red), CD29 (green)); nuclei were stained with DAPI (blue); g – result of PCR assay for episomal vector elimination and mycoplasma contamination of the cells obtained. Scale bars are 100 μm.

**Fig. 2.**
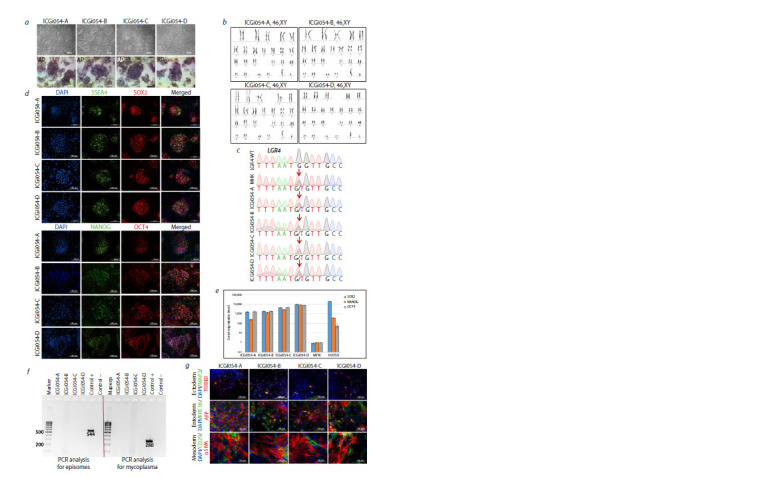
Characterisation of four patient-specific ICGi054-A, ICGi054-B, ICGi054-C, and ICGi054-D iPSC lines. a – colony morphology and detection of alkaline phosphatase (AP) in iPSCs; b – karyotyping of iPSC lines using DAPI banding; c – sequenograms of PCR products obtained from genomic DNA from a PD69 Parkinson’s disease patient, a healthy donor (LGR4-WT), iPSC lines, and PBMCs corresponding to positions 27377174-27377186 on human chromosome 11 (GRCh38 genome assembly). The position of the gene variant studied is LGR4 NC_000011.10:g.27377180C>A. The nucleotide substitution LGR4:c.1087G>T is indicated by the red arrow; d – immunofluorescence staining for markers of pluripotency: NANOG (green), OCT4 (red), SSEA-4 (green), SOX2 (red); nuclei stained with DAPI (blue); e – quantitative RT-PCR for pluripotency markers (OCT4, SOX2, NANOG) of derived iPSC lines, patient PBMCs and human embryonic stem cell line HUES9; f – immunofluorescence staining of spontaneously differentiated cells for markers of ectoderm (TUBB3 (red), MAP2 (green)), entoderm (keratin 18/KRT18 (green), AFP (red)) and mesoderm (αSMA (red), CD29 (green)); nuclei were stained with DAPI (blue); g – result of PCR assay for episomal vector elimination and mycoplasma contamination of the cells obtained. Scale bars are 100 μm.

All lines from both patients have a morphology characteristic of pluripotent cells, flat monolayer dense colonies with a large nuclear to cytoplasmic ratio, expressing endogenous alkaline phosphatase (Fig. 1a, 2a).

In the karyotyping of the obtained iPSC lines, 56 to 60 metaphases were analysed. From 6 to 12 chromosome spreads were obtained for each cell line, in accordance with the recommendations based on the European standards for cytogenetic and molecular cytogenetic studies of constitutive and acquired chromosomal abnormalities (Hastings et al., 2012; ISCN, 2020). The appearance of cells with the number of chromosomes ranging from 42 to 45 is the result of the loss of individual chromosomes during preparation. Since the number of such cells was low and they showed an absence of different chromosomes, we did not consider them as the formation of new subclones. It is known that cultured stem cells are characterised by aneuploidy (Menzorov et al., 2016), but monosomal cells are prone to apoptosis or less intensive proliferation, so in cell culture it is common to consider lines with more than 50 % of metaphases with a full set of chromosomes as normal. Detailed analysis showed that all lines have more than 55 % of cells with a normal diploid karyotype – 46,XX for ICGi053 (Fig. 1b) and 46,XY for ICGi054 (Fig. 2b). It should be noted that the analysis of the seven iPSC lines obtained did not reveal any metaphase spreads with structural rearrangements. Detailed information on the composition of the analysed metaphase spreads can be found in Table 3.

**Table 3.**
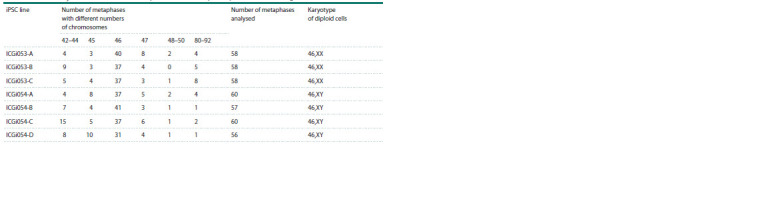
Detailed analysis of the chromosomal composition of the metaphase spreads of the resulting iPSC lines

Sanger sequencing demonstrated the presence of the c.1087G>T (rs117543292) variant of the LGR4 gene in a heterozygous state in the obtained cell lines as well as in PBMCs (Fig. 1c, 2c, the genetic variant is indicated by a red arrow). Immunofluorescence analysis of lines ICGi053-A (passage 27), ICGi053-B (passage 26), ICGi053-C (passage 27), ICGi054-A (passage 26), ICGi054-B (passage 22), ICGi054-C (passage 25) and ICGi054-D (passage 24) for pluripotency markers revealed the presence of the SSEA-4 surface antigen and the transcription factors NANOG, SOX2 and OCT4 (Fig. 1d, 2d). Quantitative real-time PCR (RTPCR) of patient PBMCs, patient-derived iPSC lines, and the control embryonic stem cell line HUES9 showed increased gene expression levels of OCT4, NANOG, and SOX2 in the iPSC lines, which was comparable to the expression level in HUES9 (Fig. 1e, 2e). The test for the ability to spontaneously differentiate into derivatives of three germ layers, which is the main confirmation of the pluripotency status of the obtained cell lines, and further immunofluorescence analysis showed the expression of markers of ectoderm (tubulin β3 (TUBB3/TUJ1), microtubule-associated protein 2 (MAP2)), entoderm (alpha-fetoprotein (AFP)), keratin 18 (KRT18)) and mesoderm (α-smooth muscle actin (αSMA), surface marker CD29) (Fig. 1f, 2f ). During cultivation, all lines underwent PCR testing for mycoplasma contamination and elimination of episomal vectors (Fig. 1g, 2g). STR analysis at 26 loci showed the identity of the iPSC lines derived from patient PBMCs (data available on request from the authors). The passports of the cell lines derived from PD58 and PD69 patients are shown in Tables 4 and 5, respectively.

**Table 4.**
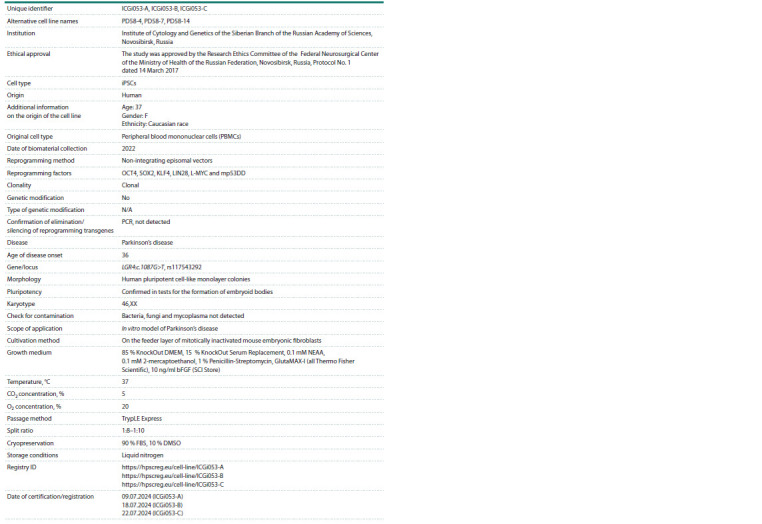
Passport of cell lines ICGi053-A, ICGi053-B, ICGi053-C

**Table 5.**
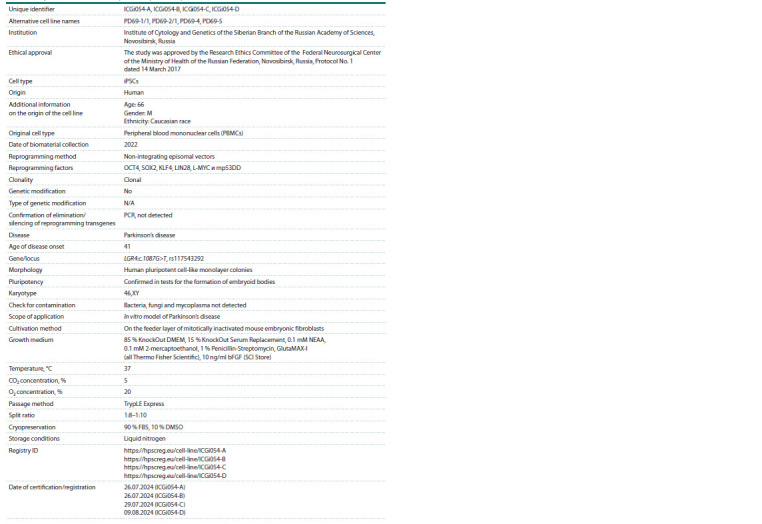
Passport of cell lines ICGi054-A, ICGi054-B, ICGi054-C, ICGi054-D

## Conclusion

As a result of reprogramming PBMCs from two Parkinson’s disease patients carrying the c.1087G>T (rs117543292) variant of the LGR4 gene, seven iPSC lines – ICGi053- A, ICGi053- B, ICGi053-C, ICGi054-A, ICGi054-B, ICGi054- C and ICGi054-D – were obtained and characterised. The cell lines obtained correspond to human pluripotent cells: they have a characteristic morphology, a normal karyotype, express pluripotency factors, are able to differentiate spontaneously into derivatives of three germ layers and have the same genetic variant LGR4:c.1087G>T as donor PBMCs. This work is the first and necessary step to study the effect of the LGR4:c.1087G>T genetic variant on the manifestation of Parkinson’s disease. The iPSC lines obtained and their differentiated derivatives represent a unique cell platform on which it will be possible in the future to study at the molecular genetic level the early pathological processes that trigger the cascade of signalling pathways leading to neuronal death, to search for targets that modulate these signalling pathways and to test new potential drugs.

## Conflict of interest

The authors declare no conflict of interest.
